# Continuous ECG monitoring versus mobile telemetry: A comparison of arrhythmia diagnostics in human- versus algorithmic-dependent systems

**DOI:** 10.1016/j.hroo.2021.09.008

**Published:** 2021-10-02

**Authors:** Mark E. Willcox, Steven J. Compton, Gust H. Bardy

**Affiliations:** ∗ Alaska Heart and Vascular Institute, Anchorage, Alaska; † University of Washington School of Medicine, Seattle, Washington; ‡ Bardy Diagnostics, Seattle, Washington

**Keywords:** Algorithms, Cardiac arrhythmias, ECG monitoring, Holter monitoring, Machine learning, MCT, Mobile cardiac telemetry

## Abstract

**Background:**

Clinicians rarely scrutinize the full disclosure of a myriad of FDA-approved long-term rhythm monitors, and they rely on manufacturers to detect and report relevant rhythm abnormalities.

**Objective:**

The objective of this study is to compare the diagnostic accuracy between mobile cardiac telemetry (MCT), which uses an algorithm-based detection strategy, and continuous long-term electrocardiography (LT-ECG) monitoring, which uses a human-based detection strategy.

**Methods:**

In an outpatient arrhythmia clinic, we enrolled 50 sequential patients ordered to wear a 30-day MCT, to simultaneously wear a continuous LT-ECG monitor. Periods of concomitant wear of both devices were examined using the associated report, which was over-read by 2 electrophysiologists.

**Results:**

Forty-six of 50 patients wore both monitors simultaneously for an average of 10.3 ± 4.4 days (range: 1.2–14.8 days). During simultaneous recording, patients were more often diagnosed with arrhythmia by LT-ECG compared to MCT (23/46 vs 11/46), *P* = .018. Similarly, more arrhythmia episodes were detected during simultaneous recording with the LT-ECG compared to MCT (61 vs 19), *P* < .001. This trend remained consistent across arrhythmia subtypes, including ventricular tachycardia (13 patients by LT-ECG vs 7 by MCT), atrioventricular (AV) block (3 patients by LT-ECG vs 0 by MCT), and AV node reentrant tachycardia (2 patients by LT-ECG vs 0 by MCT). Atrial fibrillation (AF) was documented by both monitors in 2 patients; however, LT-ECG monitoring captured 4 additional AF episodes missed by MCT.

**Conclusion:**

In a time-controlled, paired analysis of 2 disparate rhythm monitors worn simultaneously, human-dependent LT-ECG arrhythmia detection significantly outperformed algorithm-based MCT arrhythmia detection.

**Key Findings**

▪ In this trial, the real-world diagnostic accuracy of 2 extended-duration outpatient cardiac rhythm monitors were compared by asking patients to simultaneously wear both monitors.
▪ A long-term continuous electrocardiogram (LT-ECG) Carnation Ambulatory Monitor (Bardy Diagnostics, Seattle, WA) was compared to the 30-day mobile cardiac telemetry (MCT) system by Preventice Solutions (Eagan, MN).
▪ The LT-ECG picked up 3 times the number of clinically relevant arrhythmias (61 vs 19) as the MCT did in twice as many patients (23 vs 11), across a broad spectrum of arrhythmias including ventricular tachycardia, atrioventricular (AV) block, AV node reentrant tachycardia, atrial tachycardia, and atrial fibrillation over the same time period in the same patients.
▪ Fundamental differences in monitor processing exist between LT-ECG and MCT systems, with LT-ECG systems using human-based detection while MCT systems use algorithmic-based detection.
▪ Differences in the electrocardiogram quality, P-wave morphology, and clinical context provided in the reports may explain the improved specificity of the LT-ECG system.
▪ These findings indicate that not all external monitors are equal, and we hope differences highlighted in this study prompt further comparative analyses and appropriate scrutiny of artificial intelligence–based detection.

## Introduction

After a half-century of ambulatory electrocardiogram (ECG) monitoring, physicians rarely have had insight into the large set of hidden variables that affect the quality of the ECG report upon which their medical decisions depend. Those variables extend from circuit board design to electrode design and placement, signal processing, embedded algorithms, analysis software and heuristics, data transmission methods, and, most critically, the presence or absence of human readers of the digitized data either in part or in entirety.

A “gold-standard” monitor should be both sensitive in its ability to capture relevant rhythms and specific enough that the tracings and the relevant context provided to the reader (onset, offset, and heart rate trends) allow for accurate rhythm diagnosis.

In the last 10 years there has been an explosion of both medical grade (ie, US Food and Drug Administration [FDA]-approved) and commercial grade ambulatory cardiac monitoring technologies (eg, watches, clothing, etc). Much of the industry’s focus has been on ease of use, duration of recording,^1^^,^^2^ and means of report delivery to the physician (ie, “real-time” vs not).^3^

Lost in the weeds is that a monitor that fails to capture and report significant arrhythmias may cause harmful clinical misdirection, and thus a comparative understanding of diagnostic accuracy and arrhythmia detection between monitors is of utmost importance. The presumption that “an ECG monitor is an ECG monitor” has been refuted in prior prospective case studies where patients served as their own control and where diagnostic accuracy can be a product of multiple engineering and technician considerations that affect ECG fidelity and rhythm diagnostics.^4^^,^^5^

The 2 most common clinical methods for ambulatory monitoring are medical grade mobile cardiac telemetry (MCT) and continuous long-term ECG (LT-ECG). Both systems present patient-triggered events to the reader but differ in the quality of the tracings and context provided in the report to adjudicate these recordings. Fundamentally different methods are used between these systems to “automatically” detect rhythms. MCT utilizes algorithmic detection, which is subsequently reviewed by humans, whereas LT-ECG monitoring utilizes human review of the full disclosure supplemented by algorithmic processes. The critical difference between these 2 technologies is that a trained human reviews the entire data log with LT-ECG to capture and interpret strips, compared with MCT, where a trained human reviews the parts of the data log identified by an algorithm.

The purpose of this study is to compare diagnostic accuracy of algorithm-dependent vs human-dependent ECG monitoring in the same patient population, over the same period of time.

## Methods

Following informed consent, 50 patients whose treating physician referred the patients for 30-day MCT were enrolled to wear both MCT and continuous LT-ECG monitoring technologies simultaneously, starting February 20, 2020 and ending September 1, 2020. Patients were selected for consideration of the protocol in the routine course of clinical care by our research nurses in a sequential manner with the appropriate required medical indication for MCT. Patients were enrolled from the Alaska Heart and Vascular Institute, with each patient serving as their own control. The authors did not select participants. The 50 participants were recruited from 65 candidates, 15 of which refused participation for work, convenience, or Covid-related reasons.

This trial was performed according to the principles of Good Clinical Practice (Chapter 2 of the ICH Harmonized Tripartite Guideline for Good Clinical Practice), the Declaration of Helsinki, and laws and regulations about clinical studies. All patients provided written informed consent as established by an Institutional Review Board/Independent Ethics Committee/Research Ethics Board. Data for each study patient were de-identified and managed by the investigators.

Indications for monitoring and study enrollment included syncope, presyncope, palpitations, cryptogenic stroke or transient ischemic attack, cardiac arrest risk assessment, arrhythmia management concerns, and/or assessment of the cause of dyspnea or chest discomfort. Exclusion criteria were cognitive impairment, a sternal or left chest incision within 6 weeks of enrollment, and/or nursing mothers during the course of ECG recording to maximize compliance with electrode placement and monitor adhesion.

**Figure 1:**
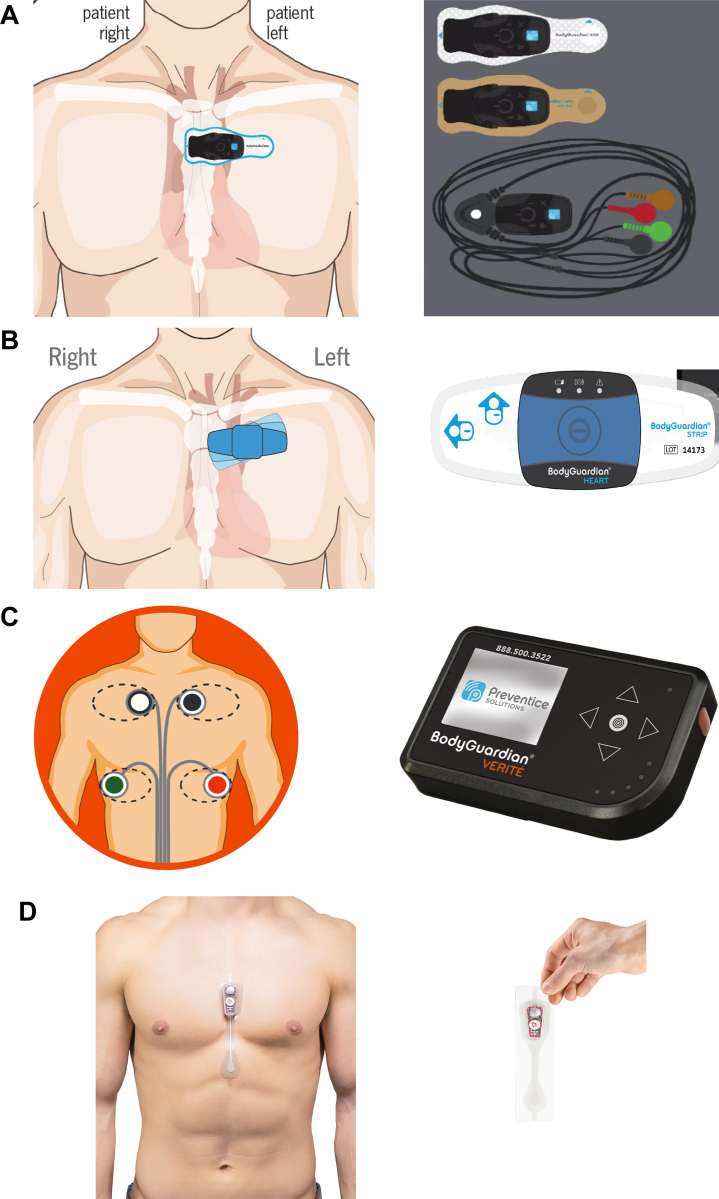
Mobile cardiac telemetry (MCT) / cardiac event monitor (CEM) devices worn (all Preventice Solutions, Eagan, MN): **A:** BioGuardian Mini Plus MCT/CEM, used in 38 patients; **B:** BodyGuardian Heart MCT/CEM, used in 5 patients; **C:** BodyGuardian Verite MCT/CEM, used in 3 patients. **D:** Long-term continuous electrocardiogram monitor (Carnation Ambulatory Monitor; Bardy Diagnostics, Seattle, WA).

Two FDA-approved cardiac ambulatory monitoring technologies were used in the study (Figure 1). The first was the Preventice MCT/cardiac event monitor (CEM) (Preventice Solutions, Eagan, MN). We chose this particular MCT device for the very simple reason that this is the only MCT manufacturer that we use in our clinic. This is related not to the MCT company but to the MCT process. The second was the Bardy Diagnostics continuous LT-ECG monitor (Bardy Diagnostics, Seattle, WA), the only LT-ECG monitor used at the Alaska Heart Institute. The Preventice MCT models employed were the BodyGuardian Mini (39 patients: 24 MCT, 15 CEM), BodyGuardian Heart (4 patients: all CEM), and BodyGuardian Verite (3 patients: 2 MCT, 1 CEM). MCT was ordered for each patient but CEM was enabled for patients that experienced insurance denials by the manufacturer at the time of MCT registration. Software analysis was performed with Paceart^TM^ and BeatLogic^TM^ software platforms (Preventice Solutions, Eagan, MN).^6^ Three different models of MCT were used from the same manufacturer, but all 3 have the same endpoint methodology employed with various electrode configurations. That said, the algorithmic diagnostic process is the same. Moreover, it is the same regardless of whether MCT defaults to CEM or not. The predominant difference between MCT and CEM is merely the 24/7 availability of a human to review algorithmic-identified events and to phone the clinic for arrhythmias that are considered critical, hence the increased cost. There is no such review during CEM unless specifically requested by the patient. The second form of ECG monitoring technology was continuous LT-ECG monitoring, sometimes called long-term “Holter,” and was restricted to the Carnation Ambulatory Monitor (Bardy Diagnostics), our only LT-ECG used. The LT-ECG monitor used a Medicare-approved independent data testing facility (IDTF) that used arrhythmia-trained human review of the entire recording period before and after a pass with Monarch^TM^ visualization software that is designed to facilitate human analysis (v. 1.1.2 for patients 1–42 and v. 1.1.3 for patients 42–50; Bardy Diagnostics).

Study participants either had the monitors placed simultaneously in the clinic or, following the onset of Covid-19, were typically mailed monitors for self-application. Devices were mailed to recipients by either the outpatient clinic or the supplying company, who provided instructions for wear. Mail-to-patient monitoring is routine with MCT and now has become more common with LT-ECG applications as remote monitoring post-Covid has increased. There are strict FDA-sanctioned instruction protocols for this process and both companies employ them, including video tutelage. Consequently, some patients did not have precise overlapping use periods.

Patients were instructed to remove each monitor at the end of the prescribed wear time at day 30 for the MCT devices and at day 14 for the LT-ECG, or as tolerated by the patient. Only periods of concomitant wear of both devices were examined for purposes of this study. Monitors were returned for processing per standard clinic guidelines and reports were prepared by each IDTF. Readers in both IDTFs were unaware of patients’ clinical trial enrollment status to ensure that readings were routine and not enhanced. All reports were received by the clinic and processed per standard operating procedure. Each IDTF report was reviewed and discussed by 2 independent electrophysiologists (MW, SC) and categorized according to the rhythm endpoints outlined below. No correlation of IDTF reports were made at the time of assessment, as reports were viewed in batches, independently and at different times. Only miscategorization of noise was corrected for study purposes by the electrophysiologists. Notably, as in clinical practice, full disclosure was not overread by electrophysiologists, who were provided with the same quality of tracings that would have been provided in the standard monitoring reporting process.

The primary endpoint of this trial was the diagnostic yield of significant arrhythmia findings during the time both monitors were worn simultaneously as reported by each IDTF. There was no clinical pre-selection for the type of possible arrhythmia findings.

Significant arrhythmias in this study were predefined as follows: atrial fibrillation (AF) of 10-second duration or longer, atrial flutter (AFL) of 10-second duration, atrial tachycardia (AT) of 20 beats or longer,^7^ ventricular tachycardia (VT) ≥3 beats at rates greater than 100 beats/min, Mobitz 1 or Mobitz 2 second-degree atrioventricular (AV) block, complete heart block, sinus bradycardia <30 beats/min for >30 seconds, 2:1 sinus node exit block, AV node reentrant tachycardia (AVNRT), or AV reentrant tachycardia.

### Statistical considerations

The primary endpoint, the incidence of patients with significant arrhythmias detected, will be assessed under the null hypothesis where each patient served as their own control and recordings with the 2 technologies were made simultaneously. For an 80% power to show a 25% difference in the ability to detect a significant arrhythmia of any type between the 2 technologies, with 95% confidence, we sought to enroll 50 patients. Statistical difference was tested using a paired *t* test.

## Results

Of the 50 patients enrolled, 2 failed to return monitors, 1 patient did not document time or date of use, and 1 failed to wear both monitors simultaneously for any period of time. Thus, of the remaining 46 who wore both monitors simultaneously for at least some time period, 15 (33%) were male and 31 (67%) were female. The mean age was 57.7 ± 15.8 (range, 21–83) years. The primary indications were palpitations (52%, n = 24), AF (28%, n = 13), syncope or near-syncope (22%, n = 11), chest pain (17%, n = 8), premature ventricular contractions (8%, n = 4), and unspecified tachycardia (6%, n = 3). More than 1 indication for ECG monitoring was provided in 24 of 46 (52%) patients. Indications are listed in Table 1. The average time both monitors recorded simultaneously was 10.3 ± 4.4 days (range, 1.2–14.8 days).

**Table 1:** Indications for cardiac monitoring

Indications†	Number of patients
Palpitations	24
Atrial fibrillation	13
Near-syncope or syncope	11
Chest pain	8
Premature ventricular contractions	4
Tachycardia	3
Bradycardia	2
Supraventricular tachycardia	2
Dizziness	2
Cardiac arrhythmia	2
Stroke	1
Abnormal ECG	1
Ischemic cardiomyopathy	1
Premature atrial contractions	1
Dyspnea	1

ECG = electrocardiogram.

† Twenty-two of 46 (48%) patients had only 1 indication and 24 of 46 (52%) patients had 2 or more indications.

During simultaneous recording, significant arrhythmias were diagnosed by the MCT monitor in 11 of 46 (24%) patients while continuous LT-ECG monitoring diagnosed significant arrhythmias in 23 of 46 (50%) of the patients, *P* = .018 (Table 2). Thus, in 12 of 46 patients (26%), a significant arrhythmia finding was only documented by LT-ECG and missed by MCT.

**Table 2:** Arrhythmias identified

Arrhythmia	MCT†/CEM	LT-ECG
**Patients diagnosed witharrhythmia duringsimultaneous wear**		
Ventricular tachycardia	7	13
Atrial fibrillation	2	2
Atrial flutter	1	1
Atrial tachycardia	3	11
Second-degree heart block	0	3
AVNRT	0	2
Total∗	11/46 (24%)	23/46 (50%)
**Number of arrhythmia episodesduring simultaneouswear**		
Ventricular tachycardia	13	27
Atrial fibrillation	2	6
Atrial flutter	1	1
Atrial tachycardia	3	15
Second-degree heart block	0	9
AVNRT	0	3
Total∗∗	19	61

Arrhythmia	Both	MCT/CEM	LT- ECG
**Overlap of detected arrhythmia episodes during simultaneous wear**			
Ventricular tachycardia	12	1	15
Atrial fibrillation	2	0	4
Atrial flutter	1	0	0
Atrial tachycardia	3	0	12
Second-degree heart block	0	0	9
AVNRT	0	0	3
Total	18	1	43

AVNRT = atrioventricular node reentrant tachycardia; CEM = cardiac event monitor; ECG = electrocardiogram; MCT = mobile cardiac telemetry.

∗ Total unique patients with significant arrhythmias 11 vs 23 *P* =.018.

∗∗ Total unique arrhythmias detected 19 vs 61 *P* <.001.

† Undetected arrhythmias occurred similarly for MCT and CEM.

The total number of significant arrhythmias detected during simultaneous recording was 19 by the MCT compared with 61 by continuous LT-ECG monitoring, *P* < .001 (Table 2). Overall, 2 or more significant arrhythmias were diagnosed in 2 of the 46 MCT (4%) patients and in 9 of the 46 patient recordings (20%) from the continuous LT-ECG monitor.

**Figure 2:**
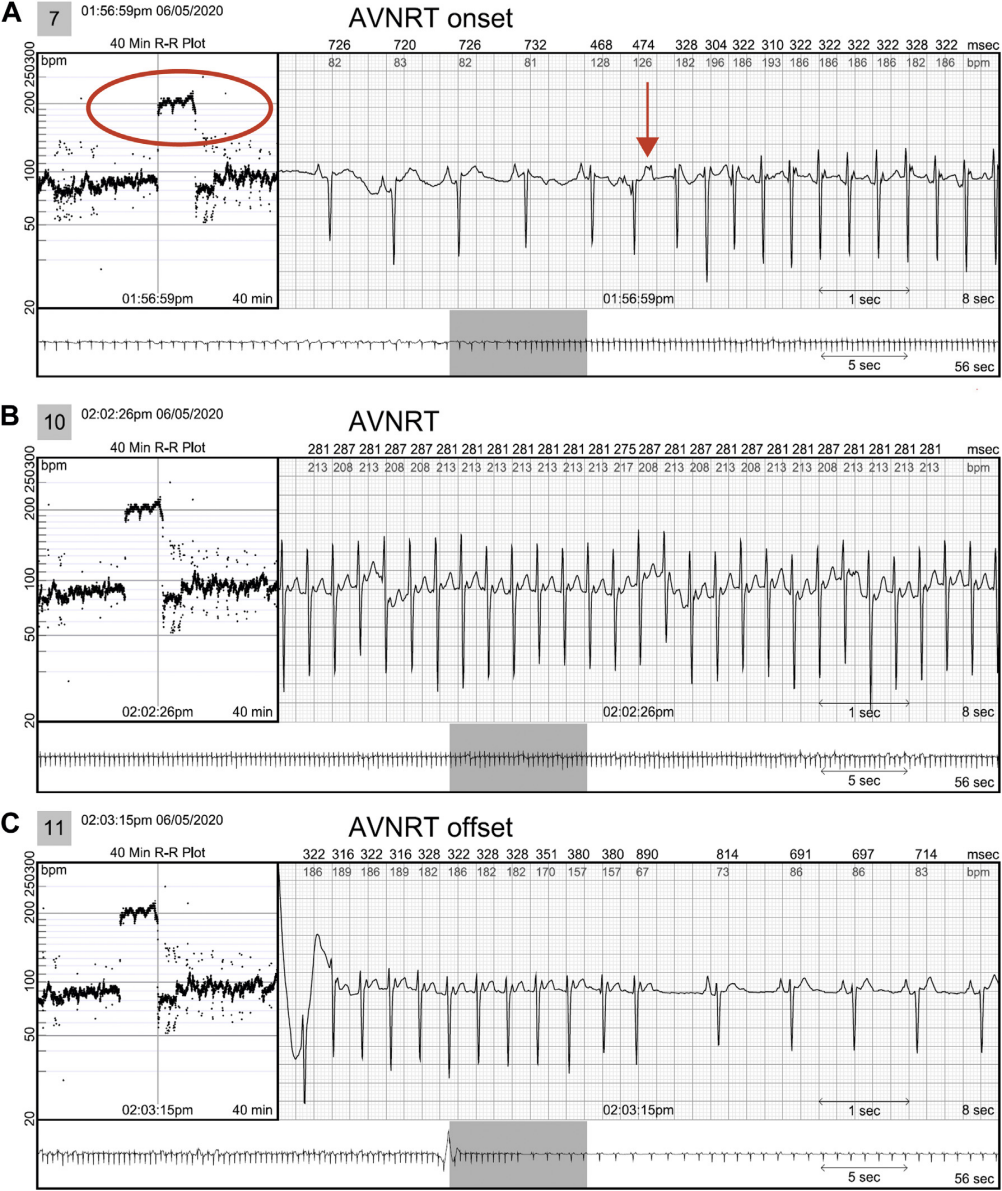

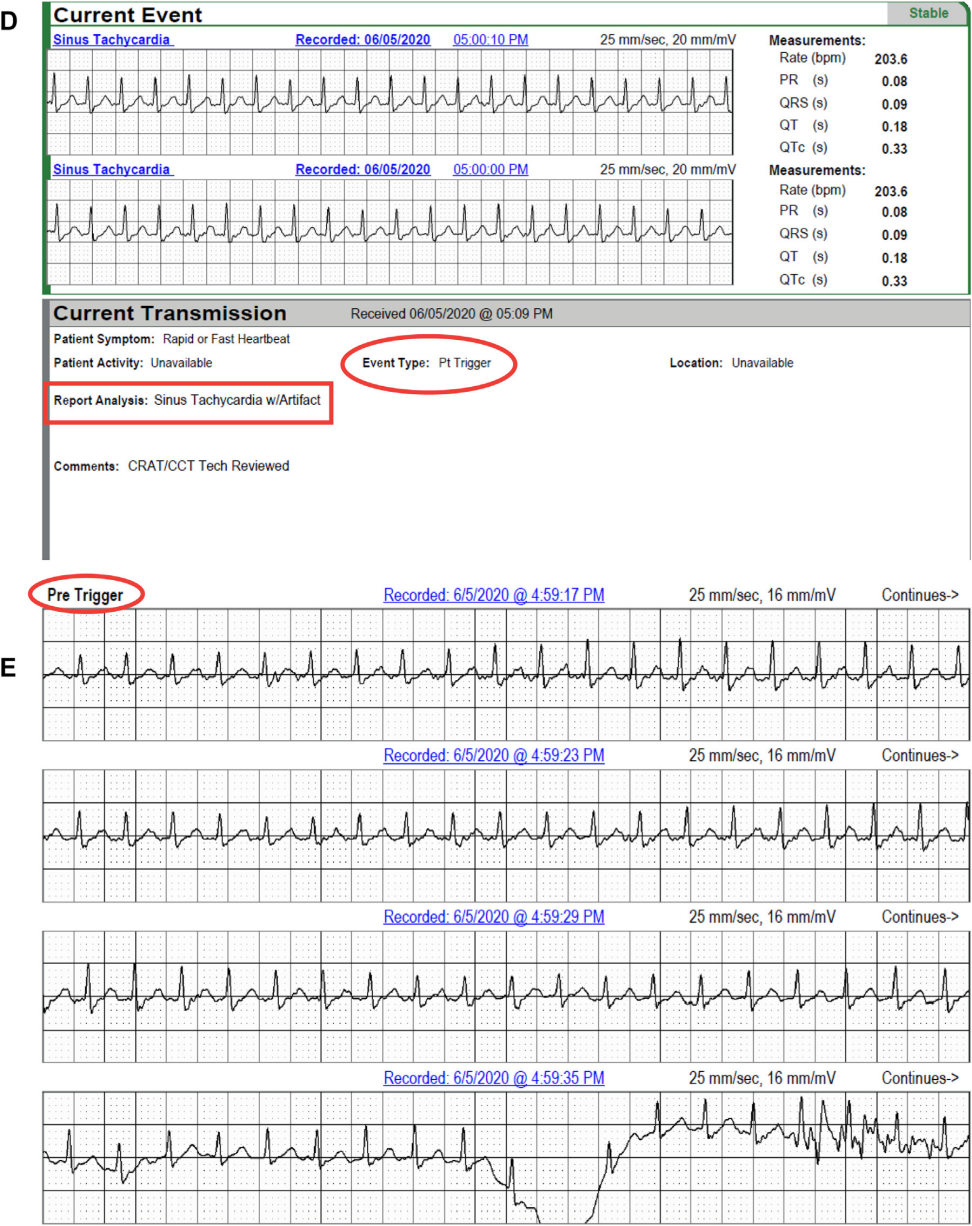

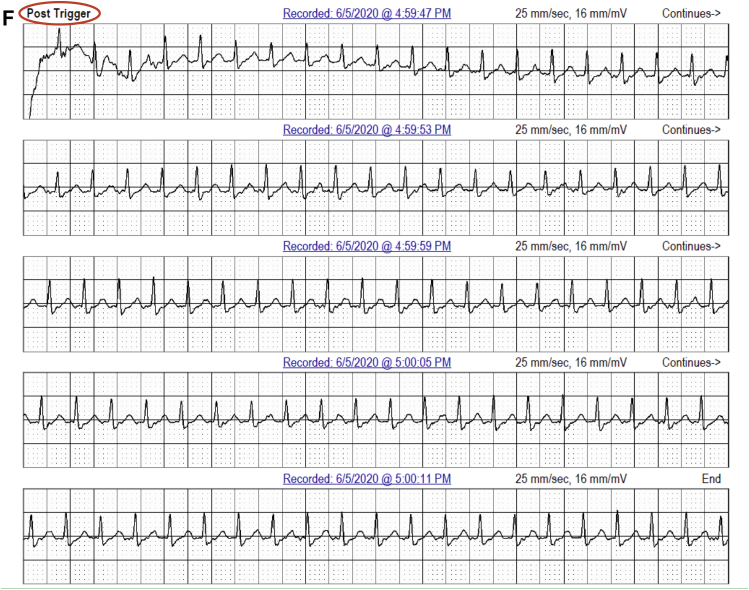
**A–C:** Results from a 56-year-old female patient with a history of palpitations, showing a 6.3-minute episode of atrioventricular node reentrant tachycardia (AVNRT) at 215 beats/min subsequently confirmed as AVNRT by electrophysiology study. **A:** Onset of the AVNRT. Note second premature atrial contraction (*red arrow*) conducts over the slow pathway (long PR interval) followed by an echo beat at the terminus of the QRS seen in every beat thereafter. Note rapid rise and fall in heart rate in the R-R plot (*red oval*) characteristic of abrupt AVNRT onset and offset. **B:** Continuation of AVNRT episode. **C:** Offset of AVNRT with classic termination with a retrograde P wave. **D–F:** Simultaneous recordings from the mobile cardiac telemetry (MCT) recorder from the same patient’s electrocardiograms shown in panels A–C. **D:** MCT provided a diagnosis of “sinus tachycardia” at a rate of 203 beats/min as a consequence of a patient trigger during a symptomatic episode of fast heartbeat (see red circle and red rectangle). No diagnosis of AVNRT was made. **E:** Additional pretrigger episode strips provided in the MCT report (*red ellipse*). **F:** Post-trigger strips (*red ellipse*) do not provide an offset of this episode.

**Figure 3:**
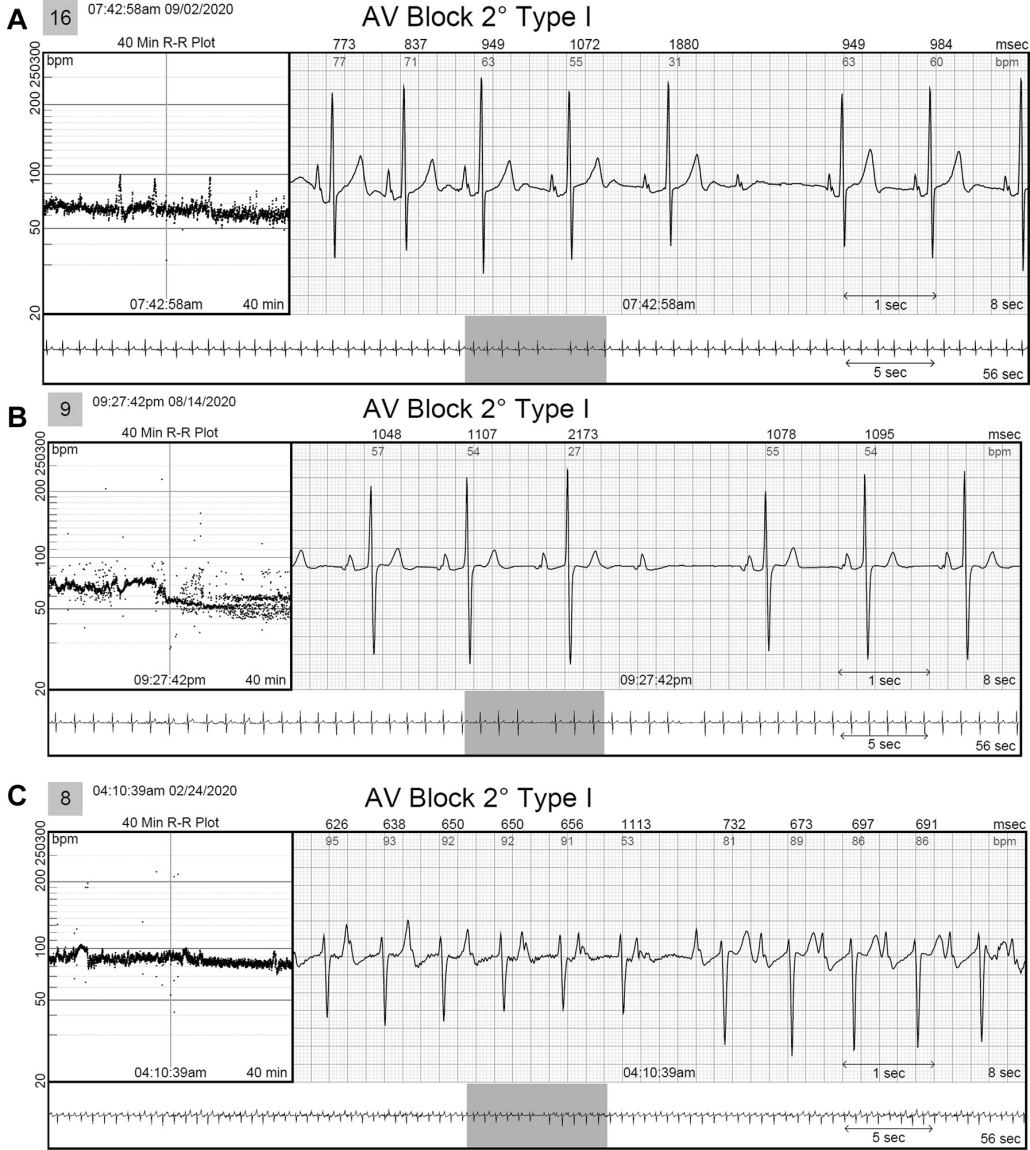
Three different patients had second-degree atrioventricular (AV) block Mobitz I recorded by the long-term continuous electrocardiogram (ECG). None of these episodes was captured on the simultaneously recorded mobile cardiac telemetry (MCT) in any of these patients. **A:** A 30-year-old woman with a history of cardiac arrhythmias, a bicuspid aortic valve, and aortic stenosis presented with chest pain and palpitations. This patient also had ventricular tachycardia (VT) missed by MCT. **B:** A 67-year-old man with a history of dyspnea and bradycardia, seen for palpitations; MCT missed the AV block but did capture the VT, as did the long-term continuous ECG. **C:** A 70-year-old woman with a history of premature ventricular contractions seen for increased dyspnea on exertion and chest tightness as well as chest radiation therapy for breast cancer. This patient had 3 episodes of AV block and 3 episodes of VT, all missed by MCT.

**Figure 4:**
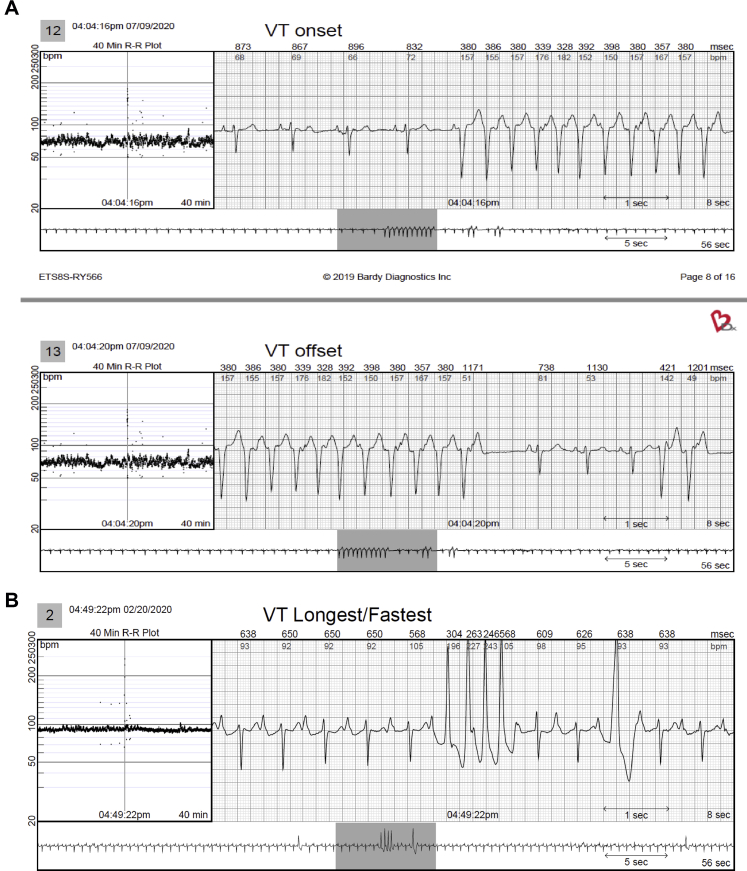

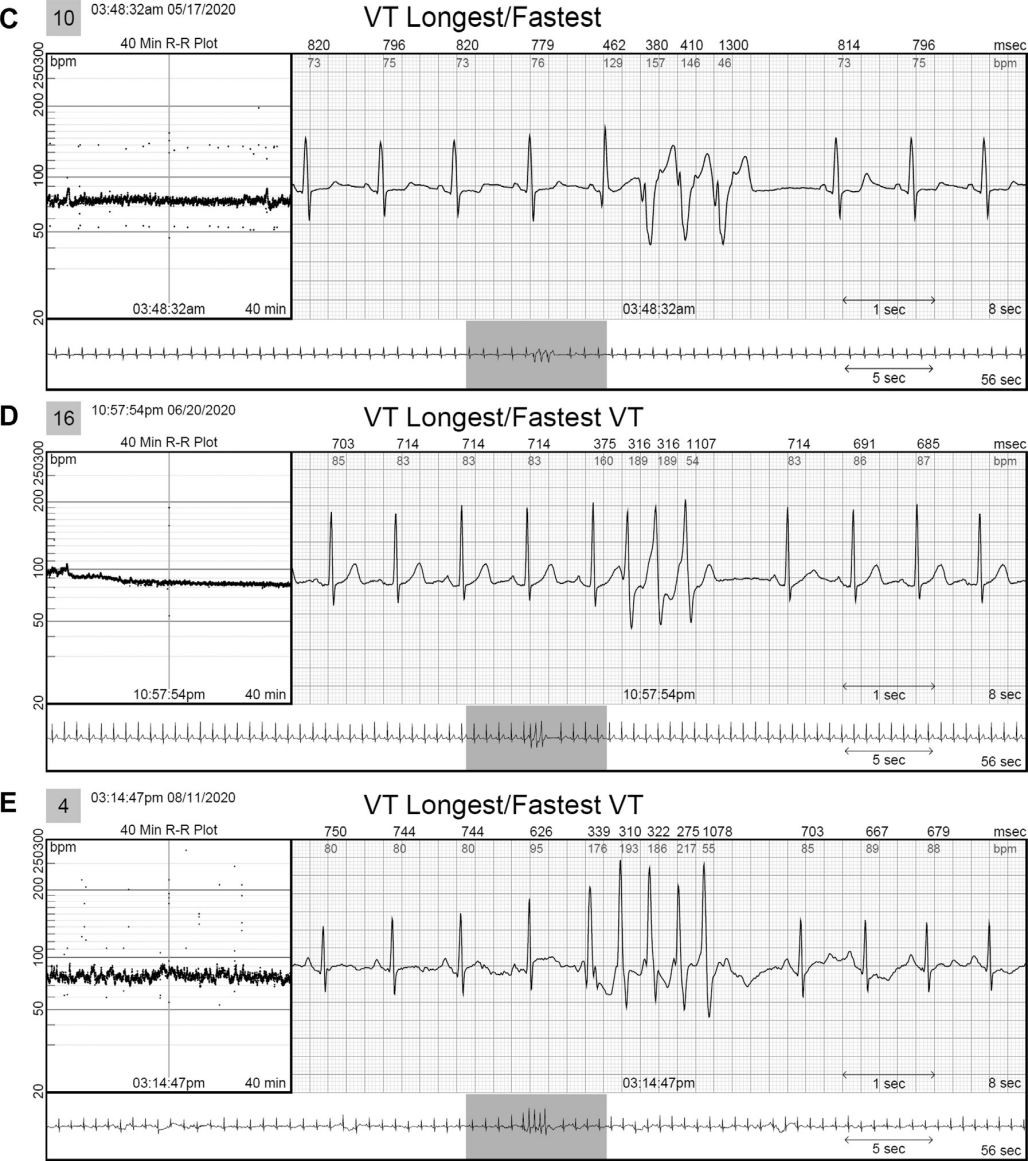

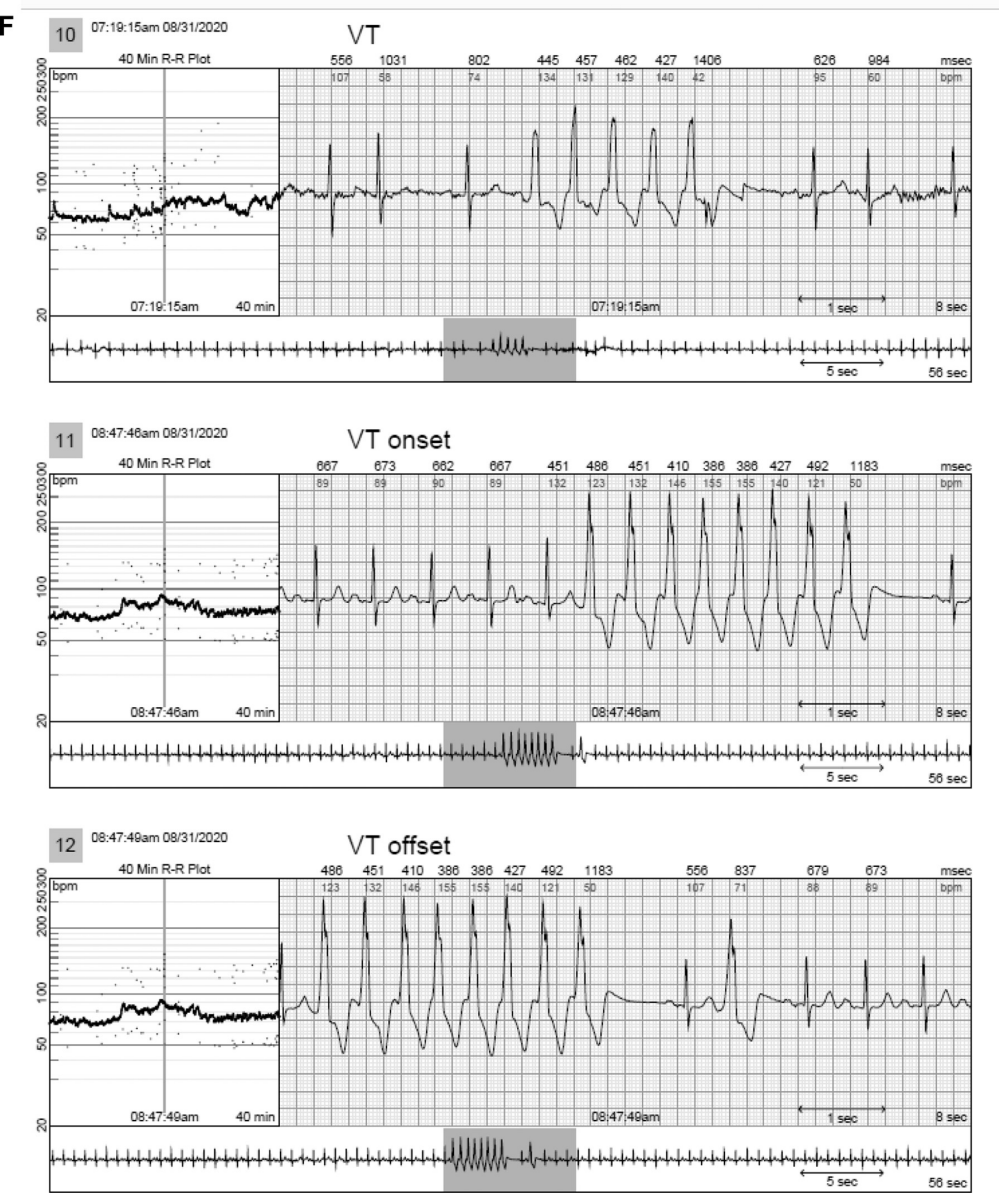
Examples of ventricular tachycardia (VT) that were only identified by the continuous long-term electrocardiography monitor and not seen on the mobile cardiac telemetry report. **A:** A 74-year-old woman with a history of palpitations and ventricular tachycardia had a 12-beat run of ventricular tachycardia (VT) at 150 beats/min. Note initiation with a short PR and ventriculoatrial dissociation and intermittent retrograde conduction. **B:** A 70-year-old woman with a history of premature ventricular contractions with a 4-beat run of VT. **C:** A 57-year-old man with a past history of persistent atrial fibrillation had a 3-beat run of VT. **D:** A 65-year-old woman with a history of syncope had a 3-beat run of VT. **E:** A 64-year-old woman with a history of palpitations had a 5-beat run of VT. **F:** A 72-year-old woman with a history of stroke and multiple runs of VT.

**Figure 5:**
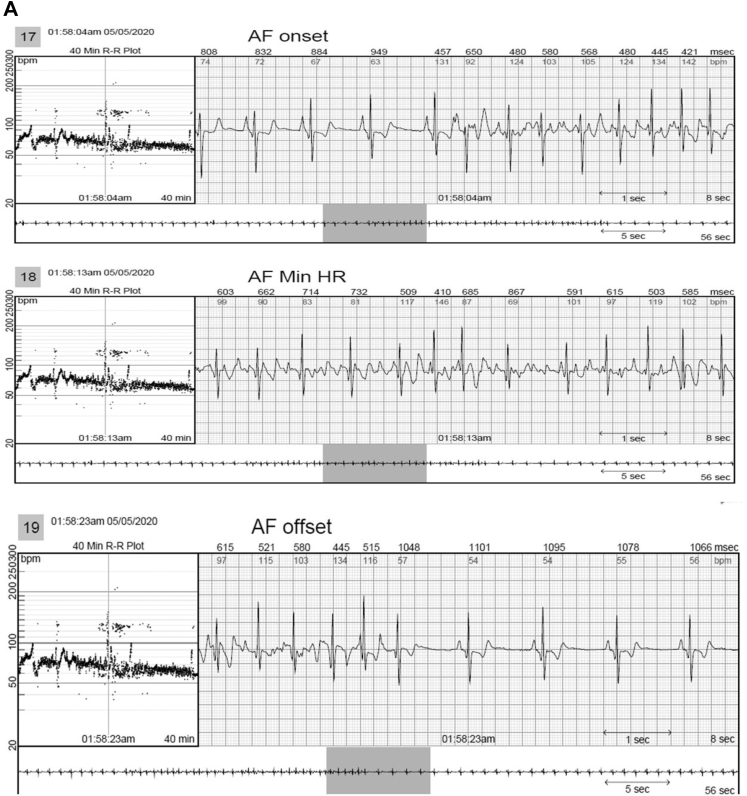

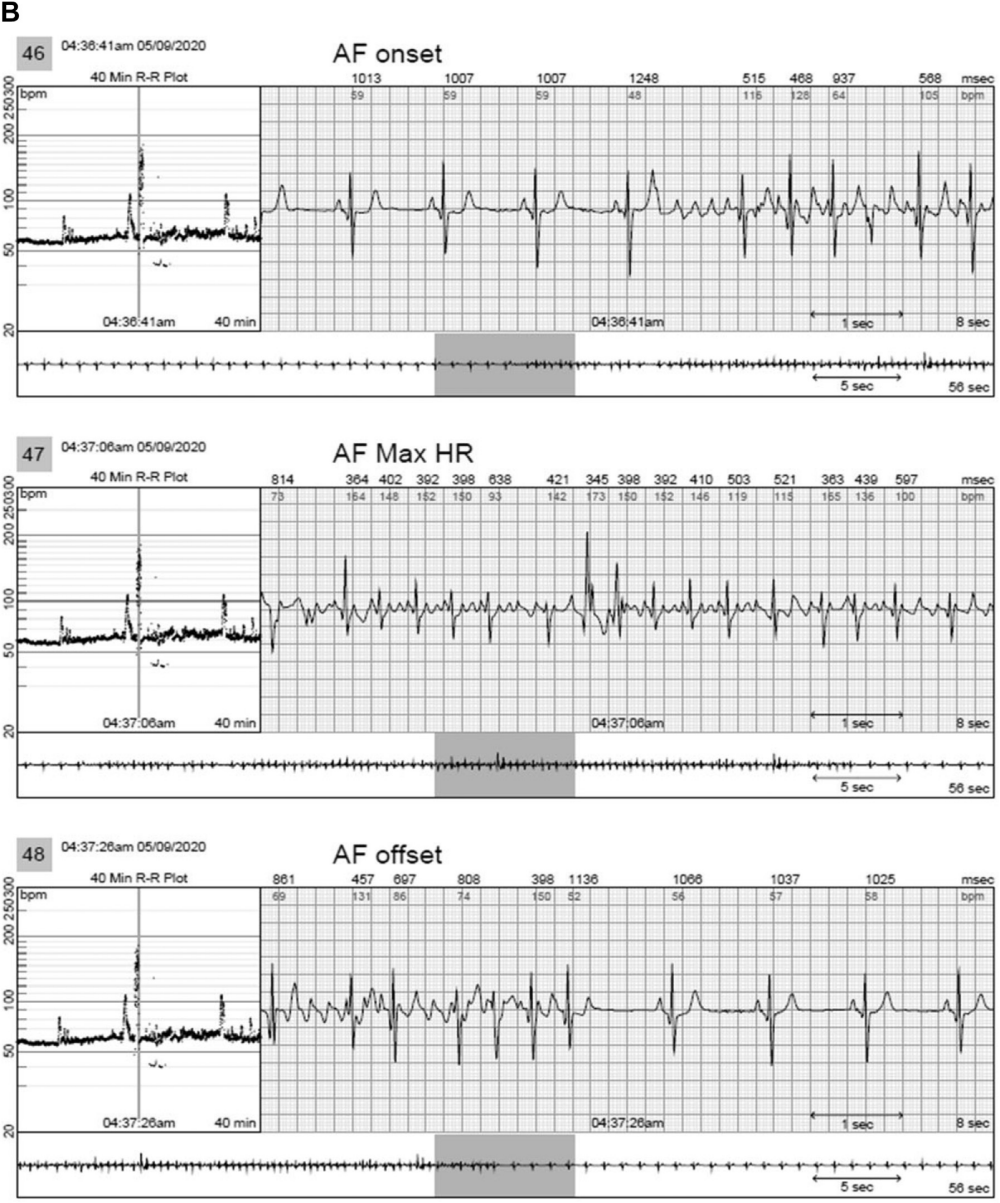

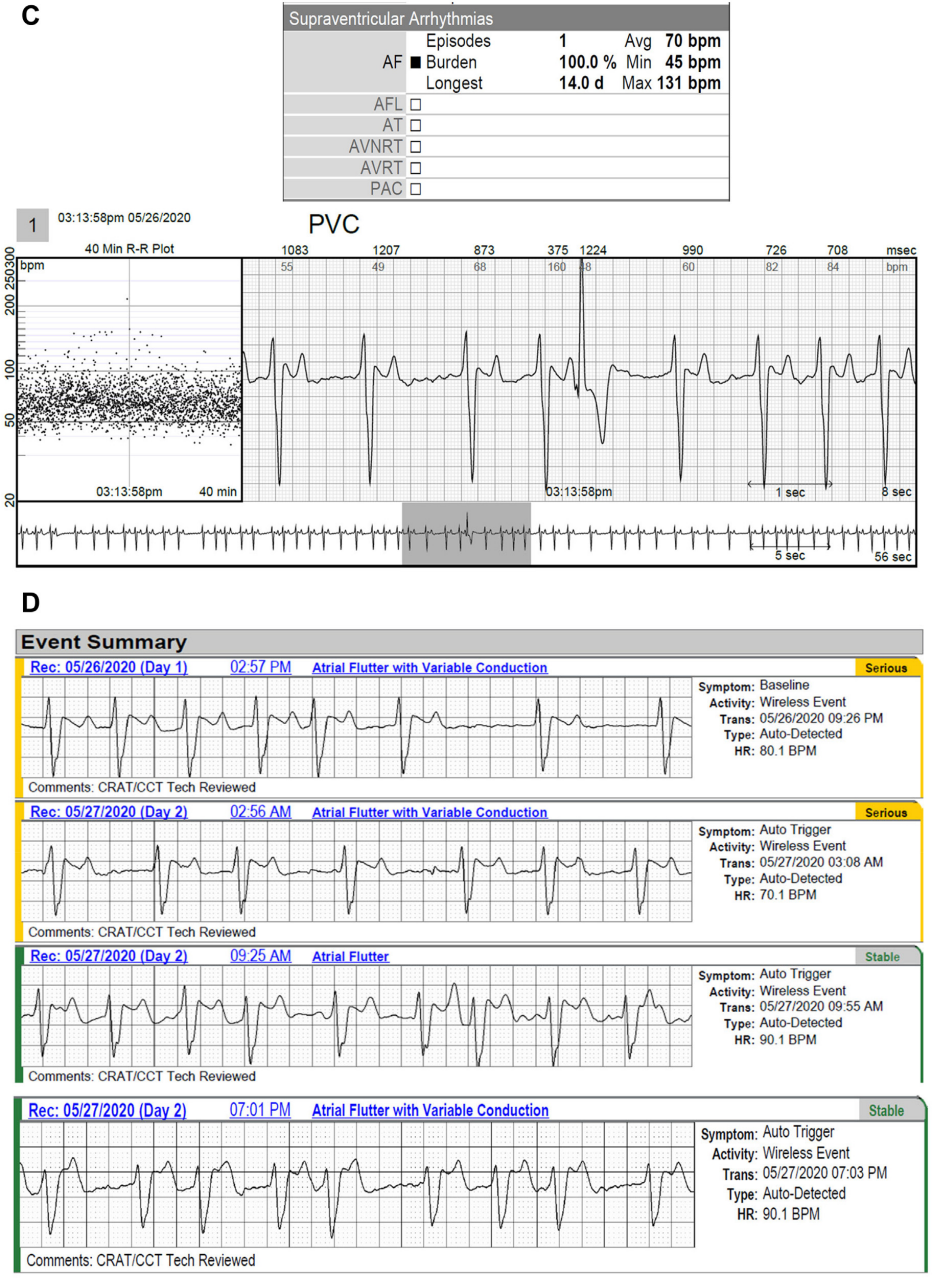
**A,B:** A 52-year-old man with a history of atrial fibrillation (AF) had 4 episodes of AF captured by long-term continuous electrocardiographic (ECG) monitoring. Images show 2 of the 4 episodes, which were brief but not captured by mobile cardiac telemetry (MCT). **C:** The continuous long-term ECG monitor documented 100% atrial fibrillation in a 76-year-old man with a history of syncope and transient ischemic attack, whereas MCT suggested atrial flutter (AFL) and AF both were present. AFL, however, was not an accurate diagnosis, as shown in **D**, representing AF rather than AFL.

In 2 patients, AVNRT captured by continuous LT-ECG monitoring was missed by MCT (in 1 patient) or misdiagnosed as sinus tachycardia (in the second patient) despite being triggered by patient activation (Table 2 and Figure 2). In 3 patients, second-degree AV block was *un*reported by MCT but captured by continuous LT-ECG monitoring (Table 2, Figure 3). VT was reported by MCT in 7 patients while VT was documented by continuous LT-ECG monitoring in 13 patients. Furthermore, only 13 VT episodes were documented by MCT, whereas 27 VT episodes were documented by continuous LT-ECG monitoring (Table 2, Figure 4). AF was documented by both types of monitor in 2 patients; however, continuous LT-ECG monitoring confirmed 4 additional episodes unreported by MCT (Table 2, Figure 5).

**Figure 6:**
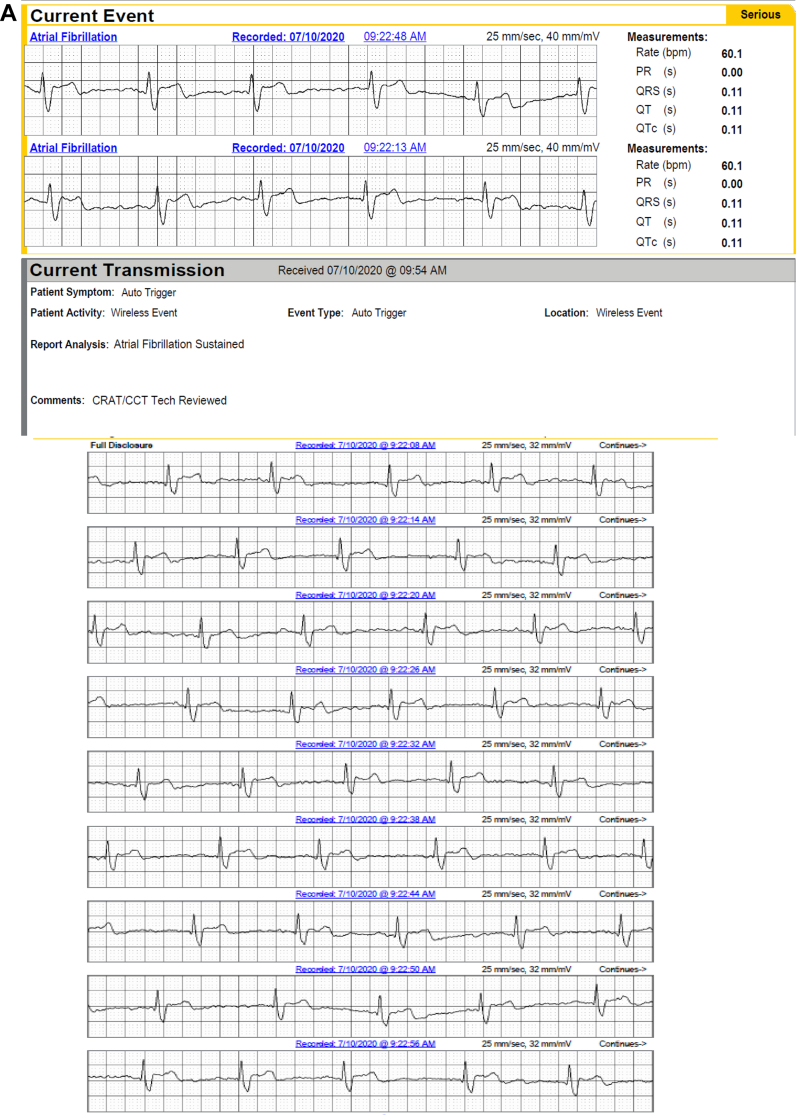

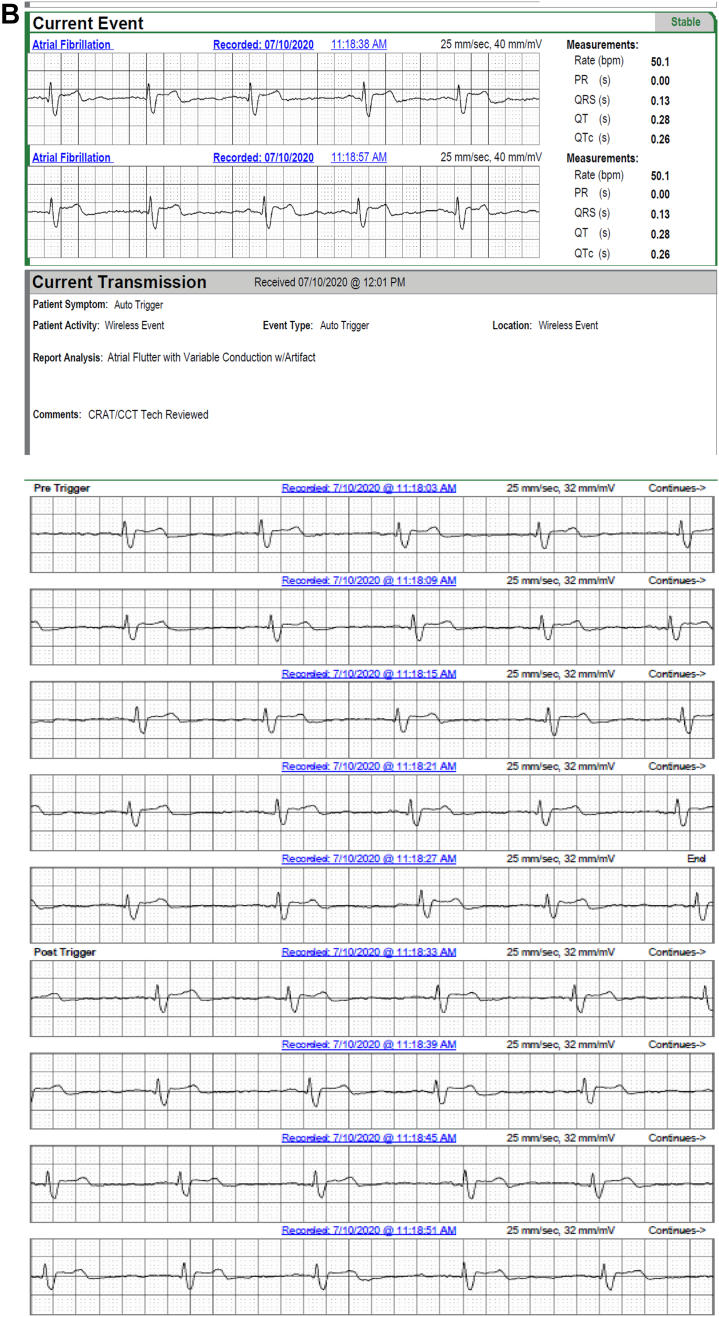
Two examples where the mobile cardiac telemetry (MCT) reported both an episode of atrial fibrillation (AF) and 1 of atrial flutter (AFL) that were determined to be false-positives upon analysis by the investigators. **A:** Sinus rhythm incorrectly called AF in the following 11 strips. **B:** Sinus rhythm called AF and AFL.

MCT was responsible for false-positive diagnoses where noise was confused both with AF on 1 recording and with AFL on another recording (Figure 6).

Time and date stamps for all strips shown in the figures use Alaska Standard Time for continuous LT-ECG monitoring and US Central Time for MCT recording, a 3-hour difference that we accounted for in the analysis.

It is notable that MCT defaulted to CEM in 43% of patients for insurance reasons, as determined by the enrolling company. Both MCT and CEM technologies use the company’s FDA-authorized hardware and software for algorithmic analysis and differ predominantly on the presence of live 24/7 telemetry, where algorithmically detected events are reviewed and communicated to ordering clinicians in “real time.” Not surprisingly, with similar detection algorithms, undetected arrhythmias compared with the LT-ECG system occurred similarly for both devices (in 58% of MCT and 42% of CEM.

## Discussion

The role of a cardiac rhythm monitor is to capture, record, and present high-quality ECG tracings for diagnosis. However, as we demonstrate here, not all monitors are equal in performing this task. Since serious adverse events can result from missed or misdiagnosed rhythms, understanding the relative accuracy of ambulatory ECG recording options is critically important. Extended duration of recording and adding clinician alerts via telemetry to a monitor certainly adds cost and may play a role in detecting and reporting rare and serious arrhythmias but cannot overcome diagnostic inaccuracy of significant arrhythmias unseen in the ECG record.

The primary outcome of this prospective ECG monitoring device comparison is that continuous LT-ECG with human oversight of the entire recording (LT-ECG) is substantially more likely to capture and report significant arrhythmias than an MCT dominantly controlled by algorithmic processes. This was true across a variety of rhythm abnormalities including VT, conduction disturbances, AT, and even supraventricular tachycardia (AVNRT). The magnitude of difference between detections is both statistically and clinically relevant, with continuous LT-ECG detecting significant arrhythmias in 50% of patients compared to 24% by MCT. In a quarter (26%) of the study population, continuous LT-ECG detected significant arrhythmias that MCT simply missed. Moreover, the quantity of significant arrhythmias detected was 3 times greater with continuous LT-ECG than with MCT (61 vs 19).

While it is difficult to objectify ECG tracing quality, continuous LT-ECG again outperformed MCT even over a relatively short study time frame. MCT misread artifact (noise) as AF in 1 patient and as AFL in another. These misdiagnoses were corrected by electrophysiologists reading the study, but could easily result in inappropriate anticoagulant therapy, testing, or worse. These 2 cases exemplify the difficulties algorithms have in differentiating noise from genuine cardiac signals. In 2 other patients, AVNRT was detected and diagnosed by continuous LT-ECG, whereas on MCT it was recorded as sinus tachycardia owing to the quality of tracings. One of these patients went on to an electrophysiology study that confirmed and ablated typical AVNRT, which may not have been the case if MCT alone had been used. These diagnostic failures raise concerns about algorithm-dependent diagnostic approaches.

What separates continuous LT-ECG monitoring from episodic ECG monitoring systems like MCT? Both need to overcome similar hurdles to data collection, skin adhesion, printed circuit board development, and systems correlating symptoms and rhythms. The fundamental difference, however, is how rhythms are detected and processed, pared down, and reported to clinicians. Continuous LT-ECG monitors utilize human interpretation of full disclosure, whereas MCT relies on algorithms.

The relative merits of ECG rhythm diagnostic tools have rarely been the subject of clinical investigation. The few available studies^4^^,^^5^^,^^8^ have shown significantly better results with continuous LT-ECG monitoring using human oversight. Prior studies of commercial algorithms have demonstrated frequent underdiagnosis^9^ as well as overdiagnosis^10^ of AF and AFL, with resultant inappropriate treatment, including antiarrhythmic therapies and anticoagulation, or unnecessary additional testing in 24% of patients.^10^ Further, there is a paucity of information on such devices having awareness of and diagnosis of more abstruse arrhythmias. Importantly, in the Lindow study,^9^ 36% of these algorithm errors were not corrected by the interpreting physician, leading to inappropriate treatment in 12 patients. Tsai and colleagues^11^ showed physicians to be highly prone to agree with and fail to correct the erroneous computer result in 67% of reports when results intentionally were made inaccurate by the investigators.

Finding significant events that may last mere moments, among many days of monitoring, is a daunting exercise requiring considerable time, knowledge, and sophisticated technologies. This challenge has prompted greater use of arrhythmia detection algorithms; although helpful to some degree, this strategy is limited by variations in the quality of the algorithms and artificial intelligence curation, signal processing, circuit board variables, and electrode characteristics. All of these variables remain closely held secrets. The importance of human oversight is rarely discussed aside these technical considerations.

Physicians rely on FDA approval process to ensure diagnostic accuracy of cardiac rhythm monitors. However, this process is heavily guided by out-of-date documents and databases^12^, ^13^, ^14^ and does not ensure uniform performance in real-world settings, partly as it does not include a broad range of known arrhythmias within the morass of ambulatory noise, something that humans are more capable at discerning. One of the standard FDA-approved test databases that is used to assess monitor performance is the 1977 MIT database, derived from 47 volunteers, that excluded complex rhythms such as AVNRT or AV reciprocating tachycardia with a concealed bypass tract—rhythms that were not well understood in 1977, nor were they in many subsequent databases now used for such software development.

Additionally, we did not detect improved diagnostic precision of serious arrhythmias when “real-time telemetry” was employed. Although this term connotes active human interpretation, human oversight occurs only after ECGs are delivered by the algorithms or by patient activation, which represent a minority of the recordings. Algorithms simply cannot contend with the broad array of arrhythmias that knowledgeable, arrhythmia-trained humans understand. Furthermore, data interruptions are common with MCT for a variety of reasons,^15^^,^^16^ generally leaving only half of the monitoring period subject to algorithmic analysis.

We hope that this head-to-head trial, where each patient served as their own control over an identical monitoring time, prompts regulatory agencies, payers, and clinicians to insist on diagnostic excellence. Comparative studies may be an important tool to assess the performance of rhythm monitoring platforms going forward. In our opinion, the combination of improved P-to-QRS visualization, context provided by an R-R plot graph, and human oversight is likely responsible for the differences we see between continuous LT-ECG and MCT.

### Limitations

There are several limitations worth mentioning. First, the study is relatively small and further differences may be evident with more subjects. Second, only 1 manufacturer’s MCT system was compared to 1 continuous LT-ECG monitor, limiting generalizability, and caution is urged not to extrapolate these findings to implanted monitoring systems with different recording duration and analysis. Third, multiple factors including noise play into a final report, limiting our ability to assess which specific engineering or interpretation strategies result in the aforementioned differences. Finally, the training of human-driven detection and interpretation as well as automated algorithms for rhythm detection can be improved over time. The findings of this study therefore should be considered limited to the technologies tested.

## Conclusion

This study demonstrated better diagnostic accuracy using continuous LT-ECG with human-aided interpretation as compared to MCT with current algorithmic-dependent rhythm identification. Not only did continuous LT-ECG recordings diagnose significant arrhythmias twice as frequently as algorithm-dependent MCT, but tracing quality and pre- and postarrhythmia context enabled complex diagnoses to be identified that were missed by MCT. Our findings suggest a greater need for comparative studies as new monitoring technologies are introduced, with human comparators as the gold standard.

## Acknowledgments

The authors would like to thank Karen Marsh, Facilities Manager for Alaska Heart Electrophysiology, and Karrie McLendon of Bardy Diagnostics for cooperation with Alaska Heart.

## Funding Sources

Funding for this investigator-initiated research trial was provided by an unrestricted grant to the Alaska Cardiovascular Research Foundation by Bardy Diagnostics.

## Disclosures

Dr Willcox and Dr Compton have no conflicts of interest to disclose. Dr Bardy is employed by Bardy Diagnostics, and has had equity in the company since 2013.

## Authorship

All authors attest they meet the current ICMJE criteria for authorship.

## Patient Consent

All patients provided written informed consent as established by an Institutional Review Board/Independent Ethics Committee/Research Ethics Board.

## Ethics Statement

This trial was performed according to the principles of Good Clinical Practice (Chapter 2 of the ICH Harmonized Tripartite Guideline for Good Clinical Practice), the Declaration of Helsinki, and laws and regulations about clinical studies. Data for each study patient were de-identified and managed by the investigators.
